# The latest freshwater giants: a new *Peltocephalus* (Pleurodira: Podocnemididae) turtle from the Late Pleistocene of the Brazilian Amazon

**DOI:** 10.1098/rsbl.2024.0010

**Published:** 2024-03-13

**Authors:** G. S. Ferreira, E. R. Nascimento, E. A. Cadena, M. A. Cozzuol, B. M. Farina, M. L. A. F. Pacheco, M. A. Rizzutto, M. C. Langer

**Affiliations:** ^1^ Senckenberg Centre for Human Evolution and Palaeoenvironment at the Eberhard Karls Universität Tübingen, Tübingen, Germany; ^2^ Geowissenschaften Fachbereich, Eberhard Karls Universität Tübingen, Tübingen, Germany; ^3^ Centro de Biologia Experimental (CIBEBI), Programa de Mestrado e Doutorado em Geografia, Universidade Federal de Rondônia (UNIR), Porto Velho, Brazil; ^4^ Facultad de Ciencias Naturales, Grupo de Investigación Paleontología Neotropical Tradicional y Molecular (PaleoNeo), Universidad del Rosario, Bogotá, Colombia; ^5^ Smithsonian Tropical Research Institute, Panamá, Panama; ^6^ Departamento de Zoologia, Instituto de Ciências Biológicas, Universidade Federal de Minas Gerais, Belo Horizonte, Brazil; ^7^ Department of Biology, University of Fribourg, Fribourg, Switzerland; ^8^ Swiss Institute of Bioinformatics, Fribourg, Switzerland; ^9^ Laboratório de Paleobiologia e Astrobiologia, Universidade Federal de São Carlos, Sorocaba, Brazil; ^10^ Instituto de Física, Universidade de São Paulo, São Paulo, Brazil; ^11^ Departamento de Biologia, Universidade de São Paulo, Ribeirão Preto, Brazil

**Keywords:** giant reptiles, vertebrate palaeontology, Testudines, body size, Amazon basin, megafauna

## Abstract

Overkill of large mammals is recognized as a key driver of Pleistocene megafaunal extinctions in the Americas and Australia. While this phenomenon primarily affected mega-mammals, its impact on large Quaternary reptiles has been debated. Freshwater turtles, due to the scarcity of giant forms in the Quaternary record, have been largely neglected in such discussions. Here we present a new giant podocnemidid turtle, *Peltocephalus maturin* sp. nov., from the Late Pleistocene Rio Madeira Formation in the Brazilian Amazon, that challenges this assumption. Morphological and phylogenetic analyses of the holotype, a massive partial lower jaw, reveal close affinities to extant Amazonian species and suggest an omnivorous diet. Body size regressions indicate *Pe. maturin* possibly reached about 180 cm in carapace length and is among the largest freshwater turtles ever found. This finding presents the latest known occurrence of giant freshwater turtles, hinting at coexistence with early human inhabitants in the Amazon.

## 1. Introduction

Overkill of large mammals is considered one of the main factors driving the extinction of the Pleistocene megafauna in the Americas and Australia [1], but it was not limited to mega-mammals. Although the Amazonian Miocene is famous for its gigantic reptiles [2], large Quaternary species are also known and might have been affected by anthropogenic action. Human predilection for giant turtles, for example, has been linked to body size decline in tortoises (Testudinidae) over the Late Neogene and Quaternary, and the overexploitation of island species leading to their extinction is well documented [3]. Freshwater turtles are usually overlooked, as giant species (straight carapace length >150 cm [3]) are absent from the Quaternary record. The largest freshwater turtles nowadays, *Chitra chitra* (Trionychidae) and *Podocnemis expansa* (Podocnemididae), reach no more than 140 and 110 cm, respectively [4], and only a handful of them have crossed the 150 cm threshold in the past [5], most recently and prominently during the Miocene [6]. Unlike their terrestrial and marine relatives, size disparity of freshwater turtles is less variable over time [5] and gigantic forms are rare. Here, we challenge this idea by presenting a new giant podocnemidid from the Late Pleistocene Rio Madeira Formation, Brazilian Amazon. The holotype of *Peltocephalus maturin* sp. nov. is composed of a partial lower jaw, which enabled us to establish its close affinities to the extant Amazonian *Peltocephalus dumerilianus* and infer an omnivorous diet. Age inferences pinpoint *Peltocephalus maturin* as the latest giant freshwater turtle, inhabiting the Amazonian rainforest on the fringe of human arrival.

## 2. Material and methods

### (a) Radiocarbon dating and geochemical analyses

Three bone samples were analysed at the Center for Applied Isotope Studies of the University of Georgia (USA) for radiocarbon dating using bioapatite protocols [7,8]. The samples were taken from the posterior portion of MERO.PV.H 007 by removing the superficial layers and digging into the fossilized bone. Micro-Raman spectroscopy and energy dispersive X-ray fluorescence (EDXRF) analyses of fossil fragments and sediments from the same locality were analysed to evaluate the reliability of the bioapatite dating [9]. Detailed descriptions of those analyses are presented in electronic supplementary material, file S1.

### (b) Morphology, body size and phylogenetics

The holotype MERO.PV.H 007 was digitized using an Artec Spider portable scanner. We also scanned lower jaws of the extant podocnemidids *Erymnochelys madagascariensis* (SMF 7879), *Peltocephalus dumerilianus* (SMF 40168), and *Podocnemis unifilis* (SMF 55470) with a Nikon XT H 320 µCT at the 3D Imaging Lab of the University of Tübingen, Germany. The dentaries were manually segmented, and surface models were saved as .stl files using Amira (v. 2020.2, ThermoFisher) and visualized on Blender (v. 3.4.1). Scanning parameters and Morphosource repository links to the datasets are shown in electronic supplementary material, file S1.

We modified a recent matrix of Pleurodira [10] by incorporating some characters from Evers *et al*. [11], as well as three new characters. We scored MERO.PV.H 007, excluded most non-Podocnemidoidae, using TNT v. 1.5 [12] (detailed descriptions on electronic supplementary material, file S1). We built a morphometric dataset comprising one angular (AJR) and seven linear measurements taken from the dentaries of 56 extant podocnemidids, two extant Pelomedusidae, and two extinct podocnemidids, MERO.PV.H 007 and VPPLT-979 (*Stupendemys geographica* [6]), to compare the new specimen within Podocnemididae. The linear measurements were divided by the maximal dentary lateral length (ML) to remove absolute size, log-transformed and plotted to characterize their distribution in the dataset (electronic supplementary material, figure S10). Principal component analysis (PCA) was applied to the morphometric dataset using the *prcomp* function in R [13], to visualize the main aspects of variation in the dentary of podocnemidids. We created a second dataset with dentary (MiL), lower jaw (JL), snout-to-mandibular condyle (SCm) and carapace (SCL) lengths for 354 specimens, sampling all the main Testudines lineages (electronic supplementary material, file S3) to estimate the body size of the new taxon. The four variables were analysed using two linear regression (*lm* function in R) approaches: predicting SCL from JL, which in turn was estimated from MiL (two regressions), and inferring SCm from the predicted JL, and then predicting SCL from SCm (three regressions). We obtained best fit, lower, and upper estimates using the *predict* function. Lower and upper bounds were then obtained using the bounds from previous regressions (full description in electronic supplementary material, file S1); this inflates error margins, but provides more realistic uncertainties, which inevitably result from using multiple regressions (R script for all analyses is presented in electronic supplementary material, file S7).

## 3. Results

### (a) Systematic palaeontology

Testudines Batsch, 1788 [14]

Pleurodira Cope, 1864 [14]

Podocnemididae Cope, 1868 *sensu* [15]

*Peltocephalus* Dumeril and Bribon, 1835

*Peltocephalus maturin* sp. nov.

### (b) Etymology

*Maturin* refers to the giant turtle that vomited out the universe in Stephen King's stories, which in turn was inspired by the character Stephen Maturin who, in the book *H.M.S. Surprise* of Patrick O'Brian's *Aubrey-Maturin* series, names a giant tortoise.

### (c) Holotype

**Figure 1.**
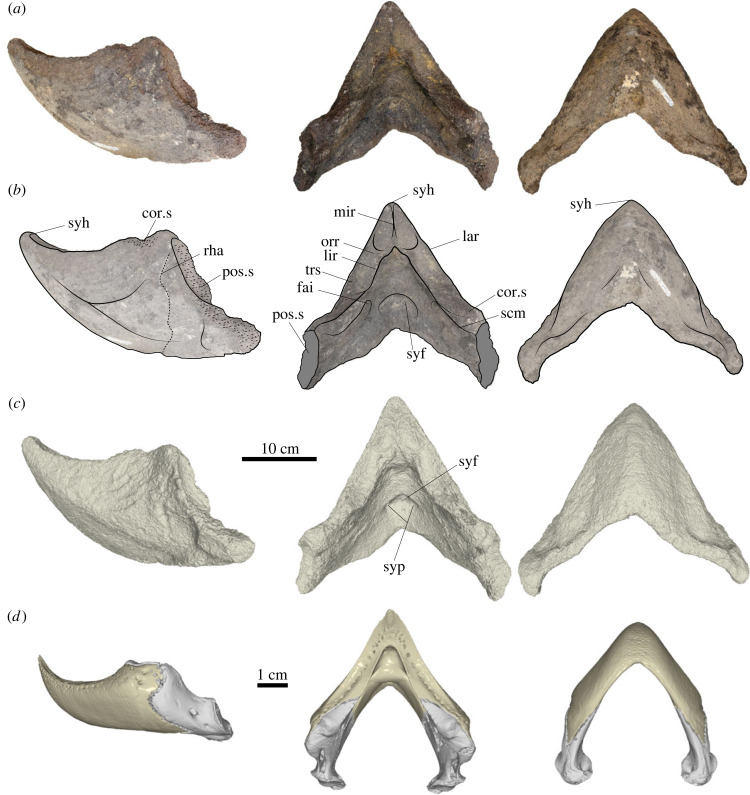
*Peltocephalus maturin* sp. nov.: photographs (*a*), outlines (*b*) and 3D renderings (*c*) of the dentary MERO.PV.H 007, and 3D renderings of *Pe. dumerilianus* lower jaw (*d*) in left lateral (left), dorsal (centre) and ventral (right) views. Abbreviations: cor.s, suture to the coronoid; fai, foramen alveolare inferius; lar, labial ridge; lir, lingual ridge; mir, midline ridge; orr, orthogonal ridge; pos.s, suture with the posterior bone; trs, triturating surface; rha, rhamphotheca posterior limit; scm, sulcus cartilagini meckeli; syf, symphyseal fossa; syh, symphyseal hook; syp, symphyseal pits.

MERO.PV.H 007 (figure 1*a–c*; electronic supplementary material, figure S7), mostly complete, massive, and fused dentaries, part of the Museu da Memória Rondoniense (MERO) collection, Porto Velho, Brazil.

### (d) Locality and horizon

MERO.PV.H 007 was collected by gold miners in the site known as Taquaras Quarry (electronic supplementary material, figure S1), in Porto Velho (Rondônia, Brazil), from unknown beds of the Rio Madeira Formation (Abunã Basin [16]), the only geological unit exposed in the quarry. That Late Pleistocene–Holocene unit was subdivided [17] into lower beds of locally laminated bioturbated claystones, with carbonized plant remains, and a greyish conglomeratic upper level, known as ‘Mucururú’ [18,19], the main auriferous and fossiliferous stratum [20]. Carbonized wood from the lower beds was dated between 46 310 and 21 310 years BP [16].

### (e) Diagnosis

*Peltocephalus maturin* is assigned to Pelomedusoides based on its fused dentaries and to *Peltocephalus* by the symphyseal hook higher than the coronoid process, the lingual platform and a small fossa with two pits on the posterior surface of the symphysis. It differs from *Podocnemis* spp. in its straight, instead of curved, labial and lingual ridges, U-shaped midline outline of the lingual ridges, well-developed symphyseal hook, transverse posterior (lingual) platform ventral to the triturating surface and the symphyseal fossa; from *Erymnochelys madagascariensis* by two pits in the symphyseal fossa, instead of one; from both *Podocnemis* spp*.* and *E. madagascariensis* in its dorsoventrally—instead of posteroventrally—sloping posterior limit of the rhamphotheca. *Peltocephalus maturin* can be distinguished from *Pe. dumerilianus* based on its much greater size, labial ridge higher than the lingual, symphyseal ridge separating left and right triturating surfaces (all these shared with *Stupendemys geographica*), and secondary ridge orthogonal to the long axis of the triturating surface, separating anterior and posterior triturating areas (autapomorphy). *Peltocephalus maturin* can be differentiated from *S. geographica* by narrow instead of expanded triturating surfaces, upcurved pointed symphyseal hook and sharp but not protruding and anteriorly V-shaped lingual ridges, instead of protruding and U-shaped.

### (f) Radiocarbon dating and geochemical analyses

Radiocarbon analyses provided ages between 14 290 ± 45 and 9060 ± 50 cal BP (detailed description in electronic supplementary material, file S1). However, high iron counts and the presence of haematite, gypsum and carbonates in the geochemical analyses indicate a poorly preserved and intensively weathered bone, and bioapatite dating has been shown to produce younger ages in warm and wet conditions [9,21]. We consider that this may be the case here, given that vertebrate fossils [17,20], palynology [19], and radiocarbon dating of sediments and carbonized trunks [16] from the Rio Madeira Formation all point to a Late Pleistocene age.

### (g) Phylogenetic and morphospace analyses, and body size estimates

**Figure 2.**
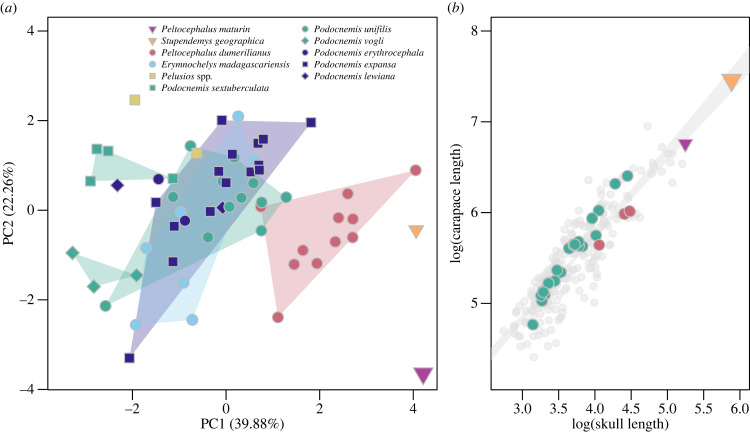
Results (*a*) of the principal component analysis of dentary linear and angular measurements and (*b*) linear regression between log-transformed skull length (SCm) and straight carapace length (SCL).

The phylogenetic analysis yielded 474 most parsimonious trees with 537 steps, the strict consensus of which shows *Peltocephalus maturin* and *Peltocephalus dumerilianus* within Erymnochelyinae as sister-taxa supported by two synapomorphies (electronic supplementary material, figure S9). Analyses of log-transformed measurements of the lower jaw (electronic supplementary material, figure S10) confirm that *Pe. maturin* differs morphologically from both *Pe. dumerilianus* and the giant Miocene podocnemidid *Stupendemys geographica*. In comparison to extant podocnemidids, the relative values of MiL, MW, TSW and TSML of *Pe. maturin* plot within the range observed for specimens of *Pe. dumerilianus*, but other metrics distinguish those species. Likewise, PCA results (PC1 = 39.88%, PC2 = 22.26%; figure 2*a*) show *Pe. maturin*, *Pe. dumerilianus*, and *S. geographica* closer on positive PC1 and the mid-range PC2, compared to *Podocnemis* spp. and *Erymnochelys madagascariensis*. These results reflect the narrow angle of the lower jaws in the group including *Pe. maturin*, as well as their smaller and broader (on the midline) triturating surfaces and higher symphyseal hook and coronoid process (electronic supplementary material, figure S7).

The linear regressions (figure 2*b*) show similar relations between the dentary and lower JL, and the latter with that of the skull (SCm) in different turtle groups (*R*² = 0.9896 and 0.9833, respectively), whereas 78.4% and 78.6% of the SCL is explained by JL and SCm, respectively. Best fitted estimates of *Pe. maturin* SCL were 170.4 mm (Approach 1) and 173.7 cm (Approach 2) with cumulative lower and upper bounds between 140.74 and 216.98 cm (detailed description in electronic supplementary material, files S1).

## 4. Discussion

Higher labial than lingual ridges and the presence of symphyseal and secondary transversal ridges support the distinctiveness of *Peltocephalus maturin* from the closely related *Peltocephalus dumerilianus*. A lingual platform, upcurved symphyseal hook, and a symphyseal fossa with two pits support the affinity of both *Peltocephalus*, as well as their distinctiveness from other podocnemidids, which is further supported by our phylogenetic and morphometric analyses. *Peltocephalus maturin* and *Pe. dumerilianus* are recovered as sister-taxa within Erymnochelyinae [15], supported by two synapomorphies (electronic supplementary material, figure S9). *Peltocephalus maturin* plots on the same quadrant as *Pe. dumerilianus* and *Stupendemys geographica* (figure 2*a*). The two latter species appear closely related in other phylogenetic analyses [10,15] and our results support this hypothesis. The PCA results also reflect less the diets of podocnemidids than their phylogenetic relations based on molecular data (e.g. [22–25]). For example, *Erymnochelys madagascariensis* clusters with the *Podocnemis* spp. albeit having a diet more like that of *Pe. dumerilianus* [26].

Considering that skull height and the proportions/complexity of the triturating surfaces adequately distinguish general feeding categories in turtles [27–30], the dentary morphology of *Pe. maturin* suggests a diet akin to that of *Pe. dumerilianus*. Although extant podocnemidids are all plant-biased omnivorous, *Pe. dumerilianus* has the highest percentage of animal items in its diet [26], commonly preying on apple snails [31]. Its simpler triturating surface reflects that habit, whereas the more herbivorous *Podocnemis* spp. show more ridged surfaces (see electronic supplementary material, figure S7). The narrow triturating surface of *Pe. maturin* is not consistent with durophagy or predominant herbivory, but its midline orthogonal ridge (absent in *Pe. dumerilianus*; electronic supplementary material, figure S7) implies a less carnivorous diet.

Size difference has been proposed to explain the coexistence of the Miocene *Caninemys tridentata* and *S. geographica* [10] and could also prevent ecological competition among extant Amazon podocnemidids, which were likely living during the Late Pleistocene [24,25]. The 27.8 cm long dentaries of *Pe. maturin* are among the largest ever found for turtles, comparable to those of the marine *Archelon ischyros*, the carapace (SCL) of which is estimated to be 221 cm (specimen NHMW-Geo 1977/1902/0001; M. Rabi 2022, personal communication). We estimated *Pe. maturin* SCL at about 180 cm, smaller than the largest known turtles, like *A. ischyros*, *S. geographica* (max SCL = 286 cm [6]), and some Quaternary tortoises [32], but larger than any Quaternary freshwater turtle from the Amazon (*Podocnemis expansa*, the largest podocnemidid SCL = 109 cm) or elsewhere (*Chitra chitra*, the largest trionychid SCL = 140 cm [4]).

Previous evidence indicated that podocnemidids attained gigantic sizes (SCL > 150 cm [3]) at least twice: Late Palaeocene [33,34] and Miocene [6,35]. This is consistent with the periods in which extreme sizes evolved in other groups of turtles [5], as well as in other reptiles, e.g. the Palaeocene giant snake *Titanoboa cerrejonensis* [36] and the giant Miocene crocodiles, such as *Purusaurus brasiliensis* [37] and *Mourasuchus pattersoni* [38]. *Peltocephalus maturin* adds giant freshwater turtles to the Pleistocene record, which already includes large squamates (e.g. *Varanus priscus*, *Wonambi naracoortensis* and *Tupinambis uruguaianensis* [39–41]) and tortoises (e.g. *Titanochelon schaefferi* and *Megalochelys atlas* [32]). Furthermore, those giant reptiles seem to have disappeared after 50–40 kyr ago [40], with testudinids showing body size decrease by the end of the Pleistocene [3].

Although explicit analyses of body size evolution in freshwater turtles are lacking, until now, no gigantic representative of this ecological group was known after the Miocene [5]. The Late Pleistocene *Peltocephalus maturin* fills this gap, hinting at the possibility that it was coeval with the early peopling of South America [42]. It has been suggested that extinctions of giant tortoises in the Late Pleistocene to Early Holocene, particularly in Australia and South America, followed a similar pattern to those of herbivorous mammals and other megafauna, and were directly related to human overexploitation [32]. There is plenty of evidence for that in the case of tortoises from remote islands, e.g. testudinids in the Turks and Caicos Islands, *ca* 1.4 kyr BP [32], and meiolaniids in Vanuatu, *ca* 3 kyr BP [43] and New Caledonia, *ca* 1.7 kyr BP [44]. Large terrestrial turtles have been part of the hominin diet since the Palaeolithic and tend to be more exploited by humans [32] because they are easier to notice and capture than smaller and freshwater turtles [45,46]. In the Amazon, some of the earliest evidence of human occupation—*ca* 12.6–11.8 kyr in Serranía La Lindosa, Colombia and *ca* 11.7–9.88 kyr in Caverna da Pedra Pintada, Brazil—is found together with both testudinid and podocnemidid remains [47–49] and, even today, the largest species are usually preferred for human consumption [50,51]. The possibility that *Pe. maturin* was part of the South American megafauna extinct by the arrival of humans is fascinating, but more data from the Late Pleistocene and Early Holocene deposits of the Amazon basin are needed to evaluate this hypothesis. In any case, what we do know is that the holotype of *Peltocephalus maturin* is one of the largest turtle dentaries ever found, revealing that a gigantic and now extinct freshwater turtle inhabited the Amazon rainforest on the fringe of human occupation of the Americas.

## Data Availability

Supporting data and code are freely available from the Dryad Repository Repository: https://doi.org/10.5061/dryad.zpc866tg2 [52] and the A*μ*CT datasets are available on Morphosource Repository Project ID: 000553087. Supplementary material is available online [53].
