# Correction: ‘The latest freshwater giants: a new *Peltocephalus* (Pleurodira: Podocnemididae) turtle from the Late Pleistocene of the Brazilian Amazon’ (2024), by Ferreira *et al.*

**DOI:** 10.1098/rsbl.2024.0151

**Published:** 2024-06-13

**Authors:** G. S. Ferreira, E. R. Nascimento, E. A. Cadena, M. A. Cozzuol, B. M. Farina, M. L. A. F. Pacheco, M. A. Rizzutto, M. C. Langer

**Affiliations:** ^1^ Senckenberg Centre for Human Evolution and Palaeoenvironment, Eberhard Karls Universität Tübingen, Tübingen, Germany; ^2^ Geowissenschaften Fachbereich, Eberhard Karls Universität Tübingen, Tübingen, Germany; ^3^ Centro de Biologia Experimental (CIBEBI), Programa de Mestrado e Doutorado em Geografia, Universidade Federal de Rondônia (UNIR), Porto Velho, Brazil; ^4^ Universidad del Rosario, Facultad de Ciencias Naturales, Grupo de Investigación Paleontología Neotropical Tradicional y Molecular (PaleoNeo), Bogotá, Colombia; ^5^ Smithsonian Tropical Research Institute, Panamá, Panama; ^6^ Departamento de Zoologia, Instituto de Ciências Biológicas, Universidade Federal de Minas Gerais, Belo Horizonte, Brazil; ^7^ Department of Biology, University of Fribourg, Fribourg, Switzerland; ^8^ Swiss Institute of Bioinformatics, Fribourg, Switzerland; ^9^ Universidade Federal de São Carlos, Laboratório de Paleobiologia e Astrobiologia, Sorocaba, Brazil; ^10^ Instituto de Física, Universidade de São Paulo, São Paulo, Brazil; ^11^ Departamento de Biologia, Universidade de São Paulo, Ribeirão Preto, Brazil

**Keywords:** giant reptiles, vertebrate palaeontology, Testudines, body size, Amazon basin, megafauna

*Biol. Lett*. 11: 20240010 (Published online 13 March 2024). (https://doi.org/10.1098/rsbl.2024.0010)

In our original publication we did not include the required ZooBank accession numbers. These are supplied here:

New species *Peltocephalus maturin* sp. nov.: urn:lsid:zoobank.org:act:4C2E8F60-A493-4B7D-A090-73C614CEF0CF

This correction should be consulted alongside the original publication as only both together fulfill the ICZN criteria.

LSIDurn:lsid:zoobank.org:pub:AD3289BF-5E8A-4C5F-97AB-F28B1C7678C2

